# Frequency of and Risk Factors for Depression among Participants in the Swiss HIV Cohort Study (SHCS)

**DOI:** 10.1371/journal.pone.0140943

**Published:** 2015-10-22

**Authors:** Alexia Anagnostopoulos, Bruno Ledergerber, René Jaccard, Susy Ann Shaw, Marcel Stoeckle, Enos Bernasconi, Jürgen Barth, Alexandra Calmy, Alexandre Berney, Josef Jenewein, Rainer Weber, Swiss HIV Cohort Study

**Affiliations:** 1 Division of Infectious Diseases and Hospital Epidemiology, University Hospital of Zurich, University of Zurich, Zurich, Switzerland; 2 Independent Researcher, HIV Practitioner, Zurich, Switzerland; 3 Division of Infectious Diseases, Cantonal Hospital St. Gallen, St. Gallen, Switzerland; 4 Division of Infectious Diseases and Hospital Epidemiology, University Hospital Basel, Basel, Switzerland; 5 Division of Infectious Diseases, Regional Hospital Lugano, Lugano, Switzerland; 6 Institute of Social and Preventive Medicine (ISPM), University of Bern, Bern, Switzerland; 7 Division of Infectious Diseases, Geneva University Hospital, Geneva, Switzerland; 8 Psychiatry Liaison Service, Lausanne University Hospital, Lausanne, Switzerland; 9 Department of Psychiatry and Psychotherapy, University Hospital of Zurich, Zurich, Switzerland; FIOCRUZ, BRAZIL

## Abstract

**Objectives:**

We studied the incidence and prevalence of, and co-factors for depression in the Swiss HIV Cohort Study.

**Methods:**

Depression-specific items were introduced in 2010 and prospectively collected at semiannual cohort visits. Clinical, laboratory and behavioral co-factors of incident depression among participants free of depression at the first two visits in 2010 or thereafter were analyzed with Poisson regression. Cumulative prevalence of depression at the last visit was analyzed with logistic regression.

**Results:**

Among 4,422 participants without a history of psychiatric disorders or depression at baseline, 360 developed depression during 9,348 person-years (PY) of follow-up, resulting in an incidence rate of 3.9 per 100 PY (95% confidence interval (CI) 3.5–4.3). Cumulative prevalence of depression during follow-up was recorded for 1,937/6,756 (28.7%) participants. Incidence and cumulative prevalence were higher in injection drug users (IDU) and women. Older age, preserved work ability and higher physical activity were associated with less depression episodes. Mortality (0.96 per 100 PY, 95% CI 0.83–1.11) based upon 193 deaths over 20,102 PY was higher among male IDU (2.34, 1.78–3.09), female IDU (2.33, 1.59–3.39) and white heterosexual men (1.32, 0.94–1.84) compared to white heterosexual women and homosexual men (0.53, 0.29–0.95; and 0.71, 0.55–0.92). Compared to participants free of depression, mortality was slightly elevated among participants with a history of depression (1.17, 0.94–1.45 vs. 0.86, 0.71–1.03, P = 0.033). Suicides (n = 18) did not differ between HIV transmission groups (P = 0.50), but were more frequent among participants with a prior diagnosis of depression (0.18 per 100 PY, 95%CI 0.10–0.31; vs. 0.04, 0.02–0.10; P = 0.003).

**Conclusions:**

Depression is a frequent co-morbidity among HIV-infected persons, and thus an important focus of care.

## Introduction

Depressive disorders are frequent among HIV-positive persons. Studies from the period before the availability of effective antiretroviral treatment (ART) reported proportions as high as 48% [1, 2], which was nearly two times higher than in HIV-negative comparison groups as shown in a meta-analysis of ten studies by Ciesla et al. [3]. Independent of HIV infection, people with depressive disorders also experience increased somatic comorbidity such as hypertension, diabetes, cardiovascular and rheumatic diseases and gastrointestinal disorders, and overall mortality [4, 5]. In the context of HIV infection, the presence of depressive disorders has been found to correlate with reduced medication adherence [6–8], more rapid progression to AIDS, and death [9–13]. Early recognition of risk factors for, and timely diagnosis of depression, and initiation of appropriate treatment, are thus important elements of any comprehensive and integrative HIV care in this high-risk population in order to improve quality of life and to prevent the negative individual and social consequences of depression.

The relative contribution of psychosocial, behavioral (including illicit and recreational drug use), and somatic (i.e., virus and ART-related) factors in the pathogenesis of depression in HIV-positive persons are not well defined. Despite suppressive ART, neuroinflammatory and neurotoxic viral effects, increased cytokine levels, and constantly elevated inflammation markers, may contribute to the development of depression [14, 15]. There have been only few studies of risk factors for and outcomes of depressive disorders since relatively well tolerated and easy to take combination ART, also including once daily single tablet regimens, became available. Some investigators found gender differences in the prevalence of severe depression with 31% among women versus 23% among men [16], while other studies did not show such differences reporting male to female ratios of 15%/15% [17] and 18%/19% [18]. Because populations studied were heterogeneous with regards to ethnicity, migration status, and non-injecting or injecting drug use, gender differences may have been masked or confounded.

Therefore, the aim of our study was to assess risk factors for incident and prevalent depression, gender differences of depression, and mortality among participants of the Swiss HIV Cohort Study (SHCS). We further sought to determine the influence of depression on suicide rates and mortality.

## Methods

### Study design and data collection

The Swiss HIV Cohort Study (SHCS) is a prospective multicenter cohort study with continuous enrollment of HIV-positive persons in Switzerland, established in 1988 [19] (www.shcs.ch). Semiannual visits with standardized data collection of demographic, psychosocial, clinical, laboratory and treatment information are conducted in the outpatient clinics of the seven cohort centers, affiliated regional hospitals, and private practitioners collaborating with the cohort centers.

We analyzed participants from the main HIV transmission groups: men who have sex with men (MSM), heterosexually infected persons (HET) and injection drug users (IDU). We excluded persons on interferon-based therapy and pregnant women. The study period starts in January 2010 when items on depression were introduced in the semiannual cohort questionnaire, and ends in July 2013.

Prior to January 2010, the SHCS database included a single item on the diagnosis of "psychiatric illness within the last 6 months" which was not further specified. Since the start of the study period in January 2010, the database of the SHCS is recording whether the diagnosis of depression was made by a psychiatrist, and whether participants with a diagnosis of depression were prescribed anti-depressive medication. The SHCS does not request the use of a specific diagnostic tool for the diagnosis of depression; psychiatrists in Switzerland use the Diagnostic and Statistical Manual of Mental Disorders (DSM) whereas Infectious Diseases specialists at SHCS centers and HIV care physicians in private practice use clinical screening questions as previously described [20]. Among the SHCS participants in our study, the diagnosis of a depressive episode was established in 63% by psychiatrists.

The SHCS was approved by the local ethical committees of the participating centers: Kantonale Ethikkommission Zürich (KEK-ZH-NR: EK-793); Ethikkommission beider Basel ("Die Ethikkommission beider Basel hat die Dokumente zur Studie zustimmend zur Kenntnis genommen und genehmigt."); Kantonale Ethikkommission Bern (21/88); Comité departmental d'éthique des specialités médicales es de médecine communautarie et de premier recours, Hôpitaux Universitaires de Genève (01–142); Commission cantonale d'éthique de la recherche sur l'être humain, Canton de Vaud (131/01); Comitato etico cantonale, Repubblica e Cantone Ticino (CE 813); Ethikkommission des Kantons St. Gallen (EKSG 12/003), and written informed consent was obtained from all participants.

### Statistical analyses

We used Stata/SE 14.0 (StataCorp, College Station, Texas, USA) for the analyses.

#### Incidence of depression

Incidence analyses included participants without a history of psychiatric disorders prior to January 2010, and free of depression at the first two semiannual follow-up visits in 2010 or thereafter. Baseline was defined as the second of the two follow-up visits. To contribute person-years (PY) of follow-up after baseline, individuals had to have at least one additional follow-up visit (i.e., a total of at least three visits after January 2010). Time was calculated from baseline until the diagnosis of depression, last follow-up visit or death, whichever occurred first. We applied uni- and multivariable Poisson regression to describe associations of incident depression with the following variables: Age (<45, 45–54, and 55+ years), injection and non-injection drug use, smoking (no, yes without cannabis, yes with cannabis), degree of physical activity (30 minutes exercise/day: none, less than once per week, and once or more per week), ability to work (medical judgment: <50%, 50–74%, and 75%+), living situation (alone single, alone but in a stable partnership, and with a partner), sexually active in the last six months (yes/no), prior AIDS-defining conditions (yes/no), virologically replicating hepatitis B (HBV) or C virus (HCV) infections, body mass index (BMI, <18.5, 18.5–24.9, 25–29.9, and 30+ kg/m^2^), CD4 cell nadir (<100, 100–199, 200–349, and 350+ cells/μL), and ART and viral suppression (not on ART, on ART with non-detectable viral load, and on ART with detectable viral load). Alcohol consumption was categorized into none, light (<20 g/day for women, <40 g/day for men), and moderate/heavy (>20 g/day for women, >40 g/day for men) according to the World Health Organization definition (http://www.who.int/publications/cra/chapters/volume1/0959-1108.pdf). All variables were time-updated and (except age) were lagged by 90 days to reduce potential reverse causality problems. To assess potential interactions between HIV transmission categories, gender and non-white ethnicity, we created an interaction term of transmission categories, gender and ethnicity: White MSM, white male HET, white female HET, white male IDU, white female IDU, non-white male and non-white female. Participants not falling in one of these categories were excluded. In preliminary analyses of antiretroviral treatment we did not find associations with time-updated/lagged regimen types by drug classes and therefore these variables were not included in the final models (all P-values >0.2, data not shown).

#### Cumulative prevalence of depression

The cumulative prevalence of depression was defined as any diagnosis of depression during follow-up among participants with at least three cohort visits during this period. Associations of cumulative prevalence of depression with the variables mentioned above were analyzed by uni- and multivariable logistic regression. For the time-updated variables we used the values prior to the diagnosis of depression or at the last follow-up visit, whichever occurred first.

#### Mortality

For mortality analyses we selected participants with at least one cohort visit after January 2010 to account for early deaths during the first year of follow-up. PY were calculated from this first visit until death or the date of the last follow-up, whichever occurred first. Differences in mortality between groups were evaluated with log-rank tests. We categorized the causes of death as HIV-related deaths, non-AIDS/HIV-related, accidents, suicides, and unknown. We also determined rates of loss to follow-up to assess the potential masking by differential rates of loss to follow-up.

#### Sensitivity analyses

We repeated the analyses without the transmission category IDU because of the strong correlation of injection drug use with HCV co-infection and several psychosocial or life-style variables such as smoking, alcohol consumption and ability to work.

## Results

### Baseline characteristics of study participants

**Fig 1:**
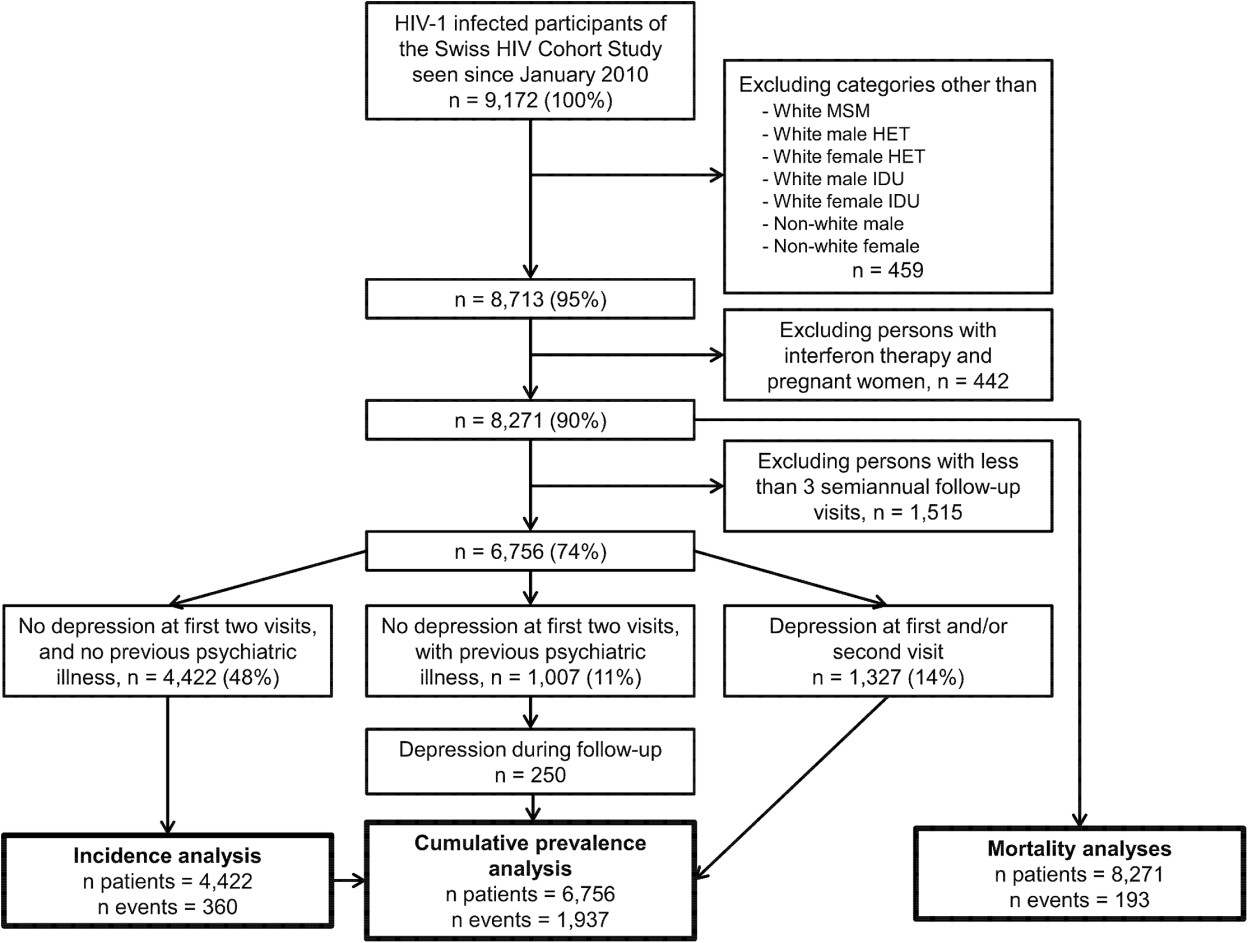
Flowchart of participant selection for the different analyses. Abbreviations: MSM, men who have sex with men; HET, heterosexually infected person; IDU, injection drug user.

The selection process for the different analyses is shown in Fig 1. For the mortality analyses we included 8,271 individuals with at least one visit after January 2010. Of these, 6,756 (82%) had at least three visits (median 4, interquartile range (IQR) 3–5), and contributed to the cumulative prevalence analysis. For the incidence analysis we included 4,422 individuals with no history of psychiatric disorders and no depression at the first two visits after January 2010.

The characteristics of participants included in the incidence and cumulative prevalence analyses are shown in Table 1. Only 39.5% and 34.7% of male and female white IDU were free of depression at both baseline visits and without previous psychiatric disorders. Persons with no history of mental health disorders at the first two visits were more likely to report light alcohol consumption, no smoking, regular physical activity, fully preserved work ability, living together with a partner, and being sexually active. They less likely had an active HCV infection, and more likely had a normal BMI. Of note, 894/1327 (67.4%) of participants with depression at baseline had a history of previous psychiatric disorders.

**Table 1:** Baseline characteristics of the 6,756 cohort participants included in the incidence and cumulative prevalence analyses.

Characteristic		Depression free at first two baseline visits without previous psychiatric disorders	Depression free at first two baseline visits with previous psychiatric disorders	Depression during period of baseline visits 1 and 2 without previous psychiatric disorders	Total
Total		4422 (65.5%)	1007 (14.9%)	1327 (19.6%)	6756 (100%)
Risk group	White MSM	1934 (69.4%)	361 (13.0%)	492 (17.7%)	2787 (100%)
	White male HET	631 (76.2%)	87 (10.5%)	110 (13.3%)	828 (100%)
	White female HET	426 (62.6%)	115 (16.9%)	140 (20.6%)	681 (100%)
	White male IDU	294 (39.5%)	219 (29.4%)	231 (31.1%)	744 (100%)
	White female IDU	138 (34.7%)	117 (29.4%)	143 (35.9%)	398 (100%)
	Non-white male	435 (78.4%)	43 (7.8%)	77 (13.9%)	555 (100%)
	Non-white female	564 (73.9%)	65 (8.5%)	134 (17.6%)	763 (100%)
Age [years]	<45	2064 (68.5%)	407 (13.5%)	542 (18.0%)	3013 (100%)
	45–54	1589 (60.7%)	446 (17.1%)	581 (22.2%)	2616 (100%)
	55+	769 (68.2%)	154 (13.7%)	204 (18.1%)	1127 (100%)
Previous psychiatric disorders	No	4422 (91.1%)	0 (0.0%)	433 (8.9%)	4855 (100%)
	Yes	0 (0.0%)	1007 (53.0%)	894 (47.0%)	1901 (100%)
Alcohol consumption	None	1895 (60.7%)	524 (16.8%)	703 (22.5%)	3122 (100%)
	Light	2280 (71.9%)	406 (12.8%)	484 (15.3%)	3170 (100%)
	Moderate/Heavy	247 (53.2%)	77 (16.6%)	140 (30.2%)	464 (100%)
Smoking	No	2682 (73.5%)	401 (11.0%)	564 (15.5%)	3647 (100%)
	Yes, without cannabis	1265 (58.9%)	370 (17.2%)	512 (23.9%)	2147 (100%)
	Yes, including cannabis	475 (49.4%)	236 (24.5%)	251 (26.1%)	962 (100%)
Activity [30 min./day]	None	1920 (60.7%)	480 (15.2%)	764 (24.1%)	3164 (100%)
	<1/week	535 (71.7%)	98 (13.1%)	113 (15.2%)	746 (100%)
	>1/week	1967 (69.1%)	429 (15.1%)	450 (15.8%)	2846 (100%)
Ability to work [%]	<50	481 (35.7%)	351 (26.0%)	517 (38.3%)	1349 (100%)
	50–74	216 (45.1%)	90 (18.8%)	173 (36.1%)	479 (100%)
	75–100	3725 (75.6%)	566 (11.5%)	637 (12.9%)	4928 (100%)
Living situation	Alone, single	1182 (58.7%)	339 (16.8%)	493 (24.5%)	2014 (100%)
	Alone, partner	512 (64.2%)	121 (15.2%)	165 (20.7%)	798 (100%)
	Not alone	2728 (69.2%)	547 (13.9%)	669 (17.0%)	3944 (100%)
Sexually active	No	1105 (52.0%)	408 (19.2%)	612 (28.8%)	2125 (100%)
	Yes	3317 (71.6%)	599 (12.9%)	715 (15.4%)	4631 (100%)
Prior AIDS diagnosis	No	3444 (66.1%)	733 (14.1%)	1030 (19.8%)	5207 (100%)
	Yes	978 (63.1%)	274 (17.7%)	297 (19.2%)	1549 (100%)
CD4 cell nadir	350+	777 (70.4%)	114 (10.3%)	213 (19.3%)	1104 (100%)
[cells/μL]	200–349	1458 (65.9%)	317 (14.3%)	439 (19.8%)	2214 (100%)
	100–199	1053 (64.4%)	257 (15.7%)	326 (19.9%)	1636 (100%)
	<100	1134 (62.9%)	319 (17.7%)	349 (19.4%)	1802 (100%)
Baseline CD4	500+	2444 (63.3%)	621 (16.1%)	798 (20.7%)	3863 (100%)
[cells/μL]	350–499	1128 (69.2%)	231 (14.2%)	271 (16.6%)	1630 (100%)
	200–349	654 (68.2%)	116 (12.1%)	189 (19.7%)	959 (100%)
	<200	196 (64.5%)	39 (12.8%)	69 (22.7%)	304 (100%)
ART and viral	On ART, VL<50 copies/mL	3419 (64.3%)	867 (16.3%)	1033 (19.4%)	5319 (100%)
suppression	On ART, VL>50 copies/mL	539 (70.1%)	74 (9.7%)	155 (20.2%)	768 (100%)
	Not on ART	464 (69.4%)	66 (9.9%)	139 (20.8%)	669 (100%)
Active HCV infection	No	4063 (69.4%)	740 (12.6%)	1055 (18.0%)	5858 (100%)
	Yes	359 (40.0%)	267 (29.7%)	272 (30.3%)	898 (100%)
Active HBV infection	No	4227 (65.6%)	960 (14.9%)	1262 (19.6%)	6449 (100%)
	Yes	195 (63.5%)	47 (15.3%)	65 (21.2%)	307 (100%)
BMI [kg/m^2^]	<18.5	200 (57.3%)	68 (19.5%)	81 (23.2%)	349 (100%)
	18.5–24.9	2611 (65.1%)	617 (15.4%)	782 (19.5%)	4010 (100%)
	25–29.9	1264 (68.2%)	264 (14.2%)	326 (17.6%)	1854 (100%)
	30+	347 (63.9%)	58 (10.7%)	138 (25.4%)	543 (100%)
Current injection	No	4391 (66.2%)	964 (14.5%)	1276 (19.2%)	6631 (100%)
drug use	Yes	31 (24.8%)	43 (34.4%)	51 (40.8%)	125 (100%)
Cocaine (non-	No	4258 (65.8%)	959 (14.8%)	1255 (19.4%)	6472 (100%)
injection)	Yes	164 (57.8%)	48 (16.9%)	72 (25.4%)	284 (100%)
Other non-injection	No	4237 (65.9%)	949 (14.8%)	1240 (19.3%)	6426 (100%)
drugs	Yes	185 (56.1%)	58 (17.6%)	87 (26.4%)	330 (100%)

Abbreviations: MSM, men who have sex with men; HET, heterosexual transmission; IDU, injection drug use; ART, antiretroviral therapy; VL, HIV-1 viral load; HBV, hepatitis B virus; HCV, hepatitis C virus; BMI, body mass index.

### Incidence of depression

**Fig 2:**
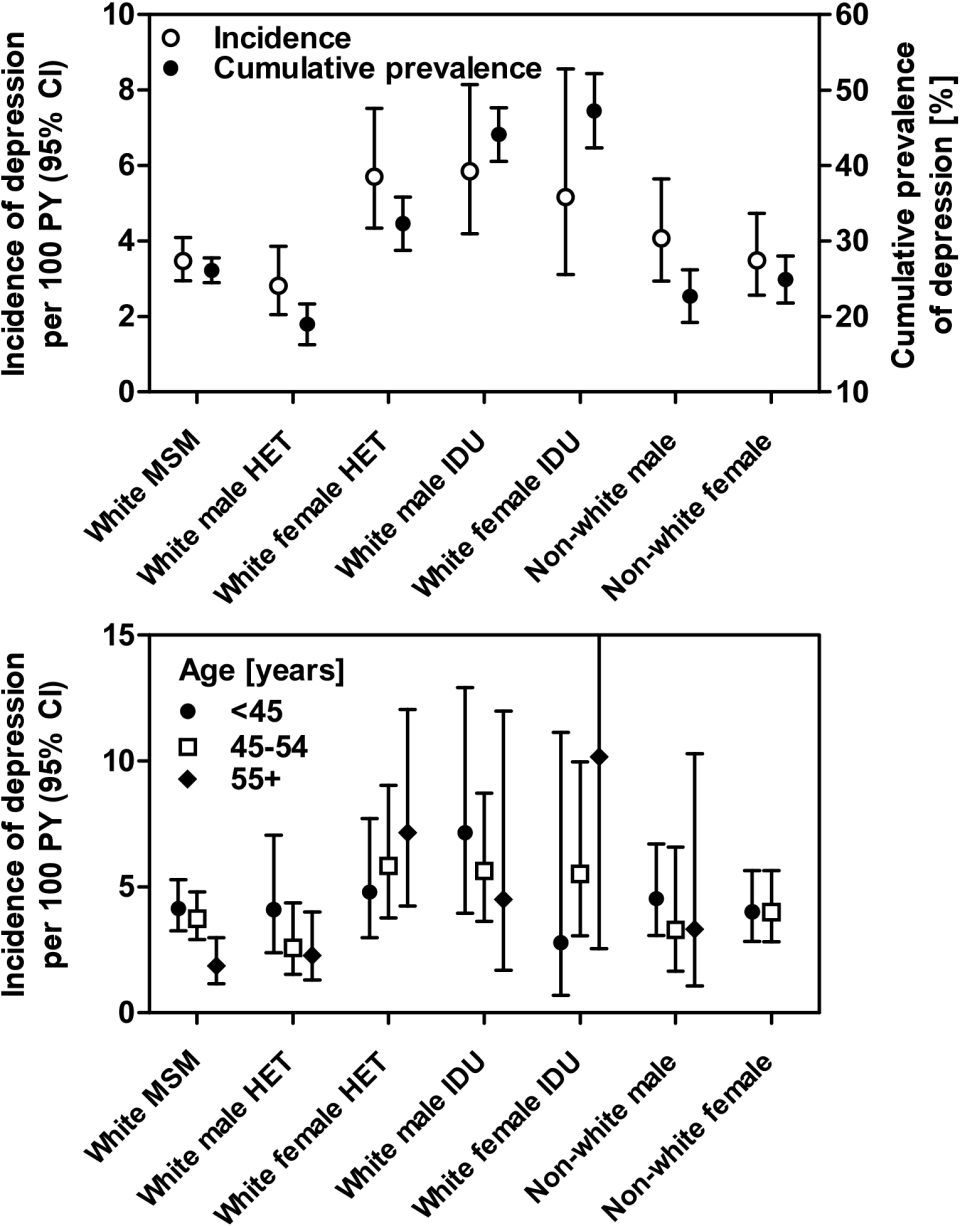
The top panel shows the incidence of depression among 4,422 individuals (360 events during 9,348 person years) without depression at the first two visits since January 2010 and cumulative prevalence of depression at the last follow-up among 6756 persons seen since January 2010 (1937 events). The bottom panel displays the incidence of depression stratified by risk and age groups. Tests for trend across age groups were MSM: P = 0.004, heterosexual men: P = 0.007, and women: P = 0.53. Abbreviations: PY, person years; MSM, men who have sex with men; HET, heterosexually infected person; IDU, injection drug user.

A new-onset depression was diagnosed in 360 of 4,422 participants without prior depressive or other psychiatric disorder during 9,348 person-years of follow-up, resulting in an incidence rate (IR) of 3.85 per 100 patient-years (95% confidence intervals (CI) 3.48–4.28). Incidence was highest for white male IDU (IR 5.85, 4.20–8.15), followed by white female HET (5.71, 4.34–7.51) and white female IDU (5.16, 3.11–8.56), and lowest for white male HET (2.81, 2.10–3.85) (Table 2 and Fig 2, top panel). Antidepressants were prescribed for 57% of participants with a depression diagnosis; of those diagnosed by psychiatrists, 70% were receiving antidepressants, whereas only 39% of those diagnosed by infectious diseases specialists or general practitioners were treated. Results of the uni- and multivariable Poisson regression analyses are shown in Table 2. Due to strong correlations between the IDU transmission category and several variables, such as alcohol consumption, smoking, ability to work, HCV and HBV co-infection as well as current injection and non-injection illicit drug use, we have omitted these variables from the multivariable model in Table 2. As supporting information we provide S1 Table with a full multivariable model demonstrating the over adjustment. In addition we performed a sensitivity analysis (S2 Table) excluding IDU due to the strong correlation of illicit drug use with HCV co-infection and several psychosocial or life-style variables such as smoking, alcohol consumption and ability to work.

**Table 2:** Poisson regression analysis of risk for incident depression among 4,422 cohort participants free of depression at the first two baseline visits and without a history of prior psychiatric disorders. Variables which correlate with IDU are omitted from the multivariable analysis.

Characteristic		Events	PY	IR	Univariable analysesIRR (95% CI)	P-value^1^	Multivariable analysisIRR (95% CI)	P-value^1^
Total		360	9348	3.9				
Risk group	White MSM	143	4117	3.5	1 (reference)	0.002	1 (reference)	0.005
	White male HET	39	1386	2.8	0.81 (0.57–1.15)		0.86 (0.60–1.24)	
	White female HET	51	894	5.7	1.64 (1.19–2.26)		1.67 (1.20–2.32)	
	White male IDU	35	598	5.8	1.68 (1.16–2.44)		1.67 (1.14–2.43)	
	White female IDU	15	291	5.2	1.49 (0.87–2.53)		1.45 (0.83–2.52)	
	Non-white male	36	885	4.1	1.17 (0.81–1.69)		1.13 (0.77–1.64)	
	Non-white female	41	1177	3.5	1.00 (0.71–1.42)		0.91 (0.62–1.33)	
Age [years]^3^	<45	165	3818	4.3	1 (reference)	0.017	1 (reference)	0.009
	45–54	143	3632	3.9	0.91 (0.73–1.14)	0.005^2^	0.89 (0.70–1.13)	0.004^2^
	55+	52	1898	2.7	0.63 (0.46–0.87)		0.60 (0.43–0.84)	
Alcohol	None	187	3969	4.7	1 (reference)	0.001		
consumption^3^ ^,^ ^4^	Light	153	4833	3.2	0.67 (0.54–0.83)			
	Moderate/heavy	20	546	3.7	0.78 (0.49–1.23)			
Smoking	No	195	5835	3.3	0.86 (0.67–1.09)	<0.001		
	Yes, without cannabis	102	2614	3.9	1 (reference)			
	Yes, including cannabis	63	899	7.0	1.80 (1.31–2.46)			
Activity	None	174	4070	4.3	1 (reference) 0.90	0.16	1 (reference) 0.88	0.13
[30 min./day]^3^ ^,^ ^4^	<1/week	42	1097	3.8	(0.64–1.25)	0.056^2^	(0.63–1.24)	0.043^2^
	>1/week	144	4180	3.4	0.81 (0.65–1.00)		0.79 (0.63–0.99)	
Ability to work [%]^3^ ^,^ ^4^	<50	57	1048	5.4	1 (reference)	0.010		
	50–74	21	430	4.9	0.90 (0.54–1.48)			
	75+ (full)	282	7869	3.6	0.66 (0.50–0.88)			
Living situation^3^ ^,^ ^4^	Alone, single	107	2423	4.4	1 (reference)	0.17	1 (reference)	0.23
	Alone, partner	46	1109	4.1	0.94 (0.67–1.33)		1.03 (0.72–1.48)	
	Not alone	207	5816	3.6	0.81 (0.64–1.02)		0.83 (0.64–1.08)	
Sexually active^3^ ^,^ ^4^	No	118	2540	4.6	1 (reference)	0.017	1 (reference)	0.027
	Yes	242	6808	3.6	0.77 (0.61–0.95)		0.75 (0.58–0.97)	
Prior AIDS	No	283	7260	3.9	1 (reference)	0.67	1 (reference)	0.88
diagnosis^3^ ^,4^	Yes	77	2088	3.7	0.95 (0.73–1.21)		1.02 (0.76–1.38)	
CD4 cell nadir	350+	74	1406	5.3	1 (reference)	0.029	1 (reference)	0.025
[cells/µL]^3^ ^,^ ^4^	200–349	119	3215	3.7	0.70 (0.53–0.94)	0.021^2^	0.68 (0.50–0.92)	0.012^2^
	100–199	84	2269	3.7	0.70 (0.51–0.96)		0.68 (0.48–0.95)	
	<100	83	2459	3.4	0.64 (0.47–0.88)		0.57 (0.39–0.83)	
ART and viral	On ART, VL <50 copies/mL	307	8168	3.8	1 (reference)	0.41	1 (reference)	0.94
suppression^3^ ^,^ ^4^	On ART, VL >50 copies/mL	26	622	4.2	1.11 (0.75–1.66)		1.04 (0.70–1.56)	
	Not on ART	27	558	4.8	1.29 (0.87–1.91)		0.95 (0.61–1.47)	
Active HCV	No	314	8620	3.6	1 (reference)	<0.001		
infection^3^ ^,^ ^4^	Yes	46	728	6.3	1.74 (1.27–2.37)			
Active HBV	No	347	8924	3.9	1 (reference)	0.40		
infection^3^ ^,^ ^4^	Yes	13	424	3.1	0.79 (0.45–1.38)			
BMI [kg/m^2^]^3^ ^,^ ^4^	<18.5	22	342	6.4	1.74 (1.12–2.70)	0.10	1.50 (0.96–2.35)	0.34
	18.5–24.9	200	5402	3.7	1 (reference)		1 (reference)	
	25–29.9	107	2824	3.8	1.02 (0.81–1.29)		1.11 (0.87–1.40)	
	30+	31	781	4.0	1.07 (0.73–1.57)		1.13 (0.76–1.68)	
Current injection	No	354	9293	3.8	1 (reference)	0.009		
drug use^3^ ^,^ ^4^	Yes	6	55	10.9	2.87 (1.30–6.36)			
Cocaine	No	339	9054	3.7	1 (reference)	0.004		
(non-injection)^3^ ^,^ ^4^	Yes	21	294	7.1	1.91 (1.23–2.96)			
Other non-injection	No	343	9030	3.8	1 (reference)	0.17		
drugs^3^ ^,^ ^4^	Yes	17	318	5.4	1.41 (0.86–2.30)			

^1^ P-values from Poisson regression unless indicated otherwise,

^2^ P-values from Poisson regression testing for trend across groups

^3^ Variable has been time-updated,

^4 ^Variable has been lagged for 90 days

Abbreviations: IR, incidence rate per 100 PY; IRR, incidence rate ratio; CI, confidence interval; PY, person years of follow-up; MSM, men who have sex with men; HET, heterosexual transmission; IDU, injection drug use; ART, antiretroviral therapy; VL, HIV viral load; HBV, hepatitis B virus; HCV, hepatitis C virus; BMI, body mass index.

Consistent among all analyses was evidence for an increased incidence of depression among white female HET with incidence rate ratios (IRR) of 1.6–1.7 when compared to MSM. Similar increases were also observed among white male IDU while there was no evidence for increased depression incidence among non-white individuals when compared to MSM (Table 2). We further found associations of incident depression with cannabis consumption and illicit drug use, both, injection and non-injection. We observed decreased incidence of depression with older age, light alcohol consumption (compared to none) throughout all models, while the associations with higher ability to work, increased physical activity and being sexually active were evident only in a subset of analyses.

The increased rates of depression for individuals with active HCV infection in the analysis of the entire cohort is difficult to interpret because of the high prevalence of replicating HCV among IDU (60%) compared to only 3–4% among the other risk groups. In the sensitivity analyses limited to non-IDU participants (S2 Table), however, elevated depression rates for persons with active HCV co-infection were confirmed (adjusted IRR 1.87, 95% CI 1.11–3.16, P = 0.019).

Separate analyses in different HIV transmission groups showed significant declines in the incidence of depression per 10 years older age for white male HET (IRR 0.68, 95% CI 0.51–0.90) and MSM (0.79, 0.67–0.93), while no such trends were seen for the other HIV transmission groups. The different patterns of incident depression with age for the various risk groups are shown in Fig 2, bottom panel.

### Cumulative prevalence of depression

Among 6,756 persons with at least three follow-up visits spanning a median of 2.94 years (IQR 2.60–3.11) since January 2010, a total of 1,937 (28.7%) experienced a depressive disorder during follow-up. The diagnosis was made by a psychiatrist in 1212/1937 (62.6%) of participants with depression, and the prescription of anti-depressive medication was more frequent when a psychiatrist was involved compared to HIV care providers (70.1% vs. 40.3%, P<0.001). Characteristics of persons with a diagnosis made by a psychiatrist did not differ from those with a depression diagnosed by other HIV care providers of the SHCS centers (all P>0.2, details not shown).

Results from the analysis of the cumulative prevalence of depression with logistic regression models are shown in Table 3. Variables associated with incident depression were generally also associated with cumulative prevalence. In addition, we found evidence for associations of being single with increased prevalence of depression, and of suppressed HIV-1 viral load under ART with decreased prevalence of depression. The association with cannabis consumption seen in the incidence analysis was not reproduced. Similar to the incidence analysis we have added a fully adjusted model and a sensitivity analysis excluding the IDU transmission category as supportive information in S3 and S4 Tables.

**Table 3:** Logistic regression analyses of factors associated with cumulative prevalence of depression by the end of the observation period. Variables which correlate with IDU are omitted from the multivariable analysis.

Characteristic		Events	Total	%	Univariable analyses OR (95% CI)	P-value^1^	Multivariable analysisOR (95% CI)	P-value^1^
Total		1937	6756	28.7				
Risk group	White MSM	728	2787	26.1	1 (reference)	<0.001	1 (reference)	<0.001
	White male HET	157	828	19.0	0.66 (0.55–0.80)		0.70 (0.59–0.85)	
	White female HET	220	681	32.3	1.35 (1.13–1.62)		1.29 (1.06–1.56)	
	White male IDU	328	744	44.1	2.23 (1.89–2.64)		1.96 (1.64–2.35)	
	White female IDU	188	398	47.2	2.53 (2.04–3.14)		2.13 (1.69–2.68)	
	Non-white male	126	555	22.7	0.83 (0.67–1.03)		0.74 (0.59–0.93)	
	Non-white female	190	763	24.9	0.94 (0.78–1.13)		0.73 (0.60–0.89)	
Age [years]^3^	<45	798	2510	31.8	1 (reference)	<0.001	1 (reference)	<0.001
	45–54	849	2868	29.6	0.90 (0.80–1.01)	<0.001^2^	0.78 (0.68–0.88)	<0.001^2^
	55+	290	1378	21.0	0.57 (0.49–0.67)		0.49 (0.42–0.59)	
Alcohol	None	1024	3180	32.2	1 (reference)	<0.001		
consumption^3^ ^,^ ^4^	Light	727	3082	23.6	0.65 (0.58–0.73)			
	Moderate/heavy	186	494	37.7	1.27 (1.04–1.55)			
Smoking	No	847	3725	22.7	0.58 (0.51–0.65)	<0.001		
	Yes, without cannabis	707	2096	33.7	1 (reference)			
	Yes, including cannabis	383	935	41.0	1.36 (1.16–1.60)			
Activity	None	1055	3127	33.7	1 (reference)	<0.001	1 (reference)	<0.001
[30 min./day]^3^ ^,^ ^4^	<1/week	189	784	24.1	0.62 (0.52–0.75)	<0.001^2^	0.67 (0.56–0.81)	<0.001^2^
	>1/week	693	2845	24.4	0.63 (0.56–0.71)		0.65 (0.58–0.73)	
Ability to work [%]^3^ ^,^ ^4^	<50	687	1402	49.0	1 (reference)	<0.001		
	50–74	221	451	49.0	1.00 (0.81–1.23)			
	75+ (full)	1029	4903	21.0	0.28 (0.24–0.31)			
Living situation^3^ ^,^ ^4^	Alone, single	681	1958	34.8	1 (reference)	<0.001	1 (reference)	<0.001
	Alone, partner	243	839	29.0	0.76 (0.64–0.91)		0.95 (0.79–1.16)	
	Not alone	1013	3959	25.6	0.64 (0.57–0.72)		0.72 (0.63–0.82)	
Sexually active^3^ ^,^ ^4^	No	831	2284	36.4	1 (reference)	<0.001	1 (reference)	<0.001
	Yes	1106	4472	24.7	0.57 (0.52–0.64)		0.61 (0.54–0.69)	
Prior AIDS	No	1508	5190	29.1	1 (reference)	0.20	1 (reference)	0.17
diagnosis^3^ ^,^ ^4^	Yes	429	1566	27.4	0.92 (0.81–1.05)		0.90 (0.77–1.05)	
CD4 cell nadir	350+	314	993	31.6	1 (reference)	0.17	1 (reference)	0.67
[cells/μL]^3^ ^,^ ^4^	200–349	643	2282	28.2	0.84 (0.72–1.00)	0.11^2^	0.97 (0.81–1.17)	0.25^2^
	100–199	472	1661	28.4	0.86 (0.72–1.02)		0.96 (0.79–1.16)	
	<100	508	1820	27.9	0.84 (0.71–0.99)		0.89 (0.72–1.10)	
ART and viral	On ART, VL <50 copies/mL	1551	5927	26.2	1 (reference)	<0.001	1 (reference)	<0.001
suppression^3^ ^,^ ^4^	On ART, VL >50 copies/mL	205	448	45.8	2.38 (1.96–2.89)		2.22 (1.81–2.72)	
	Not on ART	181	381	47.5	2.55 (2.07–3.15)		2.49 (1.97–3.15)	
Active HCV	No	1550	5856	26.5	1 (reference)	<0.001		
infection^3^ ^,^ ^4^	Yes	387	900	43.0	2.10 (1.81–2.42)			
Active HBV	No	1840	6445	28.6	1 (reference)	0.32		
infection^3^ ^,^ ^4^	Yes	97	311	31.2	1.13 (0.89–1.45)			
BMI [kg/m^2^]^3^ ^,^ ^4^	<18.5	122	306	39.9	1.62 (1.27–2.05)	<0.001	1.19 (0.92–1.54)	0.47
	18.5–24.9	1129	3883	29.1	1 (reference)		1 (reference)	
	25–29.9	500	1932	25.9	0.85 (0.75–0.96)		0.96 (0.85–1.10)	
	30+	186	635	29.3	1.01 (0.84–1.22)		1.05 (0.86–1.28)	
Current injection	No	1869	6643	28.1	1 (reference)	0.001		
drug use^3^ ^,^ ^4^	Yes	68	113	60.2	3.86 (2.64–5.65)			
Cocaine	No	1833	6492	28.2	1 (reference)	0.001		
(non-injection)^3^ ^,^ ^4^	Yes	104	264	39.4	1.65 (1.28–2.13)			
Other non-injection	No	1813	6440	28.2	1 (reference)	<0.001		
drugs^3^ ^,^ ^4^	Yes	124	316	39.2	1.65 (1.31–2.08)			

^1 ^P-values from logistic regression unless indicated otherwise,

^2^ P-values from logistic regression testing for trend across groups

^3^ Variable has been time-updated,

^4^ Variable has been lagged for 90 days

Abbreviations: OR, Odds ratio; CI, confidence interval; PY, person years of follow-up; MSM, men who have sex with men; HET, heterosexual transmission; IDU, injection drug use; ART, antiretroviral therapy; VL, HIV viral load; HBV, hepatitis B virus; HCV, hepatitis C virus; BMI, body mass index.

At the last visit, the majority of individuals (95%) was on ART and had restored immunity with median CD4 cell counts of 595 cells/μL (IQR 438–788).

### Mortality

During 20,102 patient-years, 193 of 8,271 participants died, resulting in an overall mortality of 0.96 (95% CI 0.83–1.11) per 100 patient-years (PY). Mortality was higher among white male IDU (2.34, 1.78–3.09), white female IDU (2.33, 1.59–3.39) and white male HET (1.32, 0.94–1.84), compared to white female HET (0.53, 0.29–0.95) and MSM (0.71, 0.55–0.92). The lowest rates were observed among non-white males (0.31, 0.13–0.74) and non-white female (0.28, 0.12–0.61). Compared to participants free of depression, mortality was slightly increased among participants with a history of depression (1.17, 0.94–1.45 vs. 0.86, 0.71–1.03, P log rank test = 0.033). Causes of death included 113 non-AIDS events, 21 HIV-related conditions, 6 accidents, 18 suicides, 4 narcotics overdose and in 31 participants causes were unknown.

The overall suicide rate was 0.089 (95% Cl 0.056–0.14) per 100 PY. The majority of suicides (12/18) occurred among MSM resulting in rates of 0.14 (0.082–0.25) per 100 PY vs. 0.12 (0.037–0.36) for white male HET (3/18 suicides). However, numbers were too small to support evidence for significant difference between HIV transmission groups (log rank test P = 0.30). Twelve suicides were committed by participants with prior diagnosis of depression during follow-up, resulting in a rate of 0.18 (0.10–0.31) per 100 PY vs. 0.045 (0.020–0.10) among persons without depression (log rank test P = 0.003).

Rates of loss to follow-up differed between HIV transmission groups with the highest rates for non-white males (5.1 per 100 PY), followed by white female IDU (4.9), white male IDU (4.6), non-white female HET (3.6), white female HET (3.2), and white male HET and white MSM (both 2.8, log rank test P<0.001). However, there was no difference in drop-out rates between participants with or without depression during follow-up (log rank test P = 0.69).

## Discussion

Among participants in the Swiss HIV cohort study who mostly were on successful ART and with predominantly restored cellular immunity, we found a high rate of depressive disorders in the study period between January 2010 and July 2013. Among the 4,433 participants with no documented or reported history of psychiatric disorders or depression at baseline, the incidence rate of depression was 3.9 per 100 PY of follow-up. The cumulative prevalence of depression among 6,756 participants during a median follow-up of 3 years was 28.7%. Noteworthy is the high proportion of IDU with a history of psychiatric disorders or prevalent depression at baseline which left only 39.5% and 34.7% of male and female white IDU for incidence analyses and thus limits the generalizability of findings for this patient group.

Our epidemiologic analyses cannot distinguish between causes, cofactors, or consequences of depression. Variables associated with depression were younger age, female gender, HCV co-infection, injection and non-injection drug use, cannabis and heavy alcohol consumption, low physical activity, and reduced ability to work. Of note, prevalent depression was more likely among participants not on ART, and among participants with detectable viral load while on ART.

The high incidence of depression among white female HET in our cohort, when compared to white MSM and white male HET (5.7 vs 3.5 vs 2.8 per 100 PY), and the high cumulative prevalence (32%, 26% and 19%, respectively) are noticeable. Incidence rates for white female HET are in the same range as for injection drug users with 5.8 and 5.2 per 100 PY for white male IDU and female IDU, respectively. Regarding the influence of age, we found a significant association for less depressive disorders with older age in MSM and heterosexual men, but not in women. So far, only few published studies used settings that allowed deriving incidence rates of depression, presumably because depression often has a chronic course. Kacanec et al. [21] from the Nutrition for Healthy Living (NFHL) study used a design similar to ours, selecting 225 individuals with two consecutive semiannual visits without depression. As incident depressive disorder, however, they required the next two consecutive visits with depression instead of just one as in our study. They did not exclude persons with previous depression or psychiatric disorders and, in addition, 21% and 12% of their study population were past and current injection drug users. Between visits 1 and 2 and visits 3 and 4, 22% of persons developed depression with twice as many being female (37% vs. 18% male). These numbers are substantially higher than in our study, but the difference is likely due to the different inclusion criteria with more individuals at risk for depression in the NFHL study. Regarding prevalence of depressive disorders, our findings are similar to results published by Aljassem et al. [16] who reported severe depression among 31% of women and 23% of men. In addition, we found differential associations of depressive disorder with older age, namely men having a decreasing depression rate with older age, while women had an increasing depression rate with older age. Gender differences in depressive disorders have been widely studied in non-HIV populations, and were found to be genuine, possibly, among other reasons, due to different adverse experiences in childhood, depression and anxiety disorders in childhood and adolescence, or sociocultural roles [22].

Further co-factors of depression have also been described in other studies, for example the association of depression with decreased physical activity [23], unemployment, living alone [24], smoking, or high risk alcohol use [17]. The strong association of incident and prevalent depression with HCV co-infection as observed in our study may have biochemical explanations in terms of neural dysregulation [25], platelet serotonin transporter functionality [26], or plasma apolipoprotein E deficiency [27], but it may also be an artifact of reverse causality as several studies have shown that individuals with depressive disorders might more likely acquire and transmit HIV [28, 29], and, as a consequence, are also more likely to be co-infected with HCV. Of note, injection drug users with the highest prevalence of HCV infection and various other risks for depression [30, 31] were excluded in our analyses.

Efavirenz, a frequently prescribed non-nucleoside reverse-transcriptase inhibitor, is known to have neurological side effects. We therefore performed preliminary analyses of time-updated/lagged regimen types by drug classes and by use of efavirenz but, as other studies suggest, we also did not find evidence for associations with incident depression [32, 33].

Similar to older studies from the early years of combination ART [10, 11], we found an association of depression with crude mortality, which was mainly driven by the higher mortality among IDU. In analyses excluding IDU there was no such evidence (data not shown). As expected, most suicides were preceded by an episode of depressive disorders, and the suicide rates of 140, and 120 per 100,000 PY for MSM and white heterosexual men, were substantially higher than for the Swiss general population in 2012 with 16.6 per 100,000 PY for men (http://www.bfs.admin.ch/bfs/portal/de/index/themen/14/02/04/key/01.html accessed 22 July 2014).

The strength of our study is its size with a well-established infrastructure and protocol-driven data collection including a large number of clinical, social, behavioral and laboratory variables. The analyses are based on highly representative data with the majority of HIV-positive persons in Switzerland enrolled [19]. A limitation of our analysis is the lack of information about psychotherapy which has been shown to be very effective in the treatment of depression [34]. Further, collection of information about the diagnostic tools to establish depression diagnoses has only started very recently. Finally, the inherent problems of cohort studies are residual confounding and reverse causality issues which may blur the findings. Although we have lagged time-updated variables in the incidence analyses to reduce the reverse causality problem, the study of depression, a chronic condition with varying degree of severity, will only allow to document associations and not causality.

In conclusion, the burden of depressive disorders among HIV-positive individuals in an economically wealthy environment, with mandatory health insurance and unlimited access to medical care and ART, is still substantial. Depression is nowadays one of the major co-morbidities of HIV-positive persons, and is associated with various demographic, clinical, social, and behavioral factors. While ART for HIV continues to evolve and improve, and individuals with HIV can now look forward to longer and healthier lives, HIV care providers should remain alert to early diagnosis and treatment of depressive disorders. Further, important clinical implications of our results are the need for interdisciplinary collaboration between HIV care providers, psychiatrists and other mental-health providers, the need for careful attentiveness and treatment of comorbidities or cofactors associated with depression, particularly HCV co-infection, injection and non-injection drug and alcohol use, and low physical activity. Finally, because depression is more likely among participants not on ART, and among participants with ongoing viral replication while on ART, treatment of depression is essential for successful antiretroviral therapy, and vice versa.

## Supporting Information

S1 Table Poisson regression analysis of risk for incident depression among 4,422 cohort participants free of depression at the first two baseline visits and without a history of prior psychiatric disorders. All variables are included in the multivariable model where correlations between IDU, HCV and several life-style variables make results difficult to interpret.
(DOCX)

S2 Table Sensitivity analyses: Poisson regression analysis of risk for incident depression among 3,990 non-IDU cohort participants free of depression at the first two baseline visits and without a history of prior psychiatric disorders. (DOCX)

S3 Table Logistic regression of factors associated with cumulative prevalence of depression by the end of the observation period. All variables are included in the multivariable model where correlations between IDU, HCV and several life-style variables make results difficult to interpret.
(DOCX)

S4 Table Sensitivity analyses: Logistic regression of factors associated with cumulative prevalence of depression among 5614 non-IDU participants by the end of the observation period. All variables are included in the multivariable model.
(DOCX)
