# Supplementary material for: Frequency of and Risk Factors for Depression among Participants in the Swiss HIV Cohort Study (SHCS)
Source: PLoS One. 2015 Oct 22;10(10):e0140943. doi: 10.1371/journal.pone.0140943 (PMC4619594; doi:10.1371/journal.pone.0140943)
Supplement: S1 Table — All variables are included in the multivariable model where correlations between IDU, HCV and several life-style variables make results difficult to interpret. (DOCX) [file pone.0140943.s001.docx]

**Table S1: Poisson regression analysis of risk for incident depression among 4,422 cohort participants free of depression at the first two baseline visits and without a history of prior psychiatric disorders. All variables are included in the multivariable model where correlations between IDU, HCV and several life-style variables make results difficult to interpret.**

| Characteristic | Events PY IR | Univariable analyses  IRR (95% CI) | P-value^1^ | Multivariable analysis  IRR (95% CI) | P-value^1^ |
| --- | --- | --- | --- | --- | --- |
| Total | 360 9348 3.9 |  |  |  |  |
| Risk group  White MSM  White male HET  White female HET  White male IDU  White female IDU  Non-white male  Non-white female | 143 4117 3.5  39 1386 2.8  51 894 5.7  35 598 5.8  15 291 5.2  36 885 4.1  41 1177 3.5 | 1 (reference)  0.81 (0.57-1.15)  1.64 (1.19-2.26)  1.68 (1.16-2.44)  1.49 (0.87-2.53)  1.17 (0.81-1.69)  1.00 (0.71-1.42) | 0.002 | 1 (reference)  0.87 (0.61-1.26)  1.63 (1.16-2.30)  1.01 (0.58-1.74)  0.88 (0.44-1.73)  1.15 (0.79-1.69)  0.93 (0.62-1.39) | 0.078 |
| Age [years]^3^  <45  45-54  55+ | 165 3818 4.3  143 3632 3.9  52 1898 2.7 | 1 (reference)  0.91 (0.73-1.14)  0.63 (0.46-0.87) | 0.017  0.005^2^ | 1 (reference)  0.91 (0.72-1.16)  0.61 (0.43-0.86) | 0.015  0.008^2^ |
| Alcohol consumption^3,4^  None  Light  Moderate/heavy | 187 3969 4.7  153 4833 3.2  20 546 3.7 | 1 (reference)  0.67 (0.54-0.83)  0.78 (0.49-1.23) | 0.001 | 1 (reference)  0.74 (0.59-0.94)  0.71 (0.44-1.14) | 0.024 |
| Smoking  No  Yes, without cannabis use  Yes, including cannabis use | 195 5835 3.3  102 2614 3.9  63 899 7.0 | 0.86 (0.67-1.09)  1 (reference)  1.80 (1.31-2.46) | <0.001 | 0.95 (0.73-1.23)  1 (reference)  1.62 (1.15-2.28) | 0.006 |
| Activity [30 min./day]^3,4^  None  <1/week  >1/week | 174 4070 4.3  42 1097 3.8  144 4180 3.4 | 1 (reference) 0.90 (0.64-1.25)  0.81 (0.65-1.00) | 0.16  0.056^2^ | 1 (reference) 0.92 (0.65-1.29)  0.81 (0.64-1.02) | 0.20  0.079^2^ |

| Ability to work [%]^3,4^  <50  50-74  75+ (full) | 57 1048 5.4  21 430 4.9  282 7869 3.6 | 1 (reference)  0.90 (0.54-1.48)  0.66 (0.50-0.88) | 0.010 | 1 (reference)  0.89 (0.54-1.46)  0.69 (0.49-0.97) | 0.097 |  |
| --- | --- | --- | --- | --- | --- | --- |
| Living situation^3,4^  Alone, single  Alone, partner  Not alone | 107 2423 4.4  46 1109 4.1  207 5816 3.6 | 1 (reference)  0.94 (0.67-1.33)  0.81 (0.64-1.02) | 0.17 | 1 (reference)  1.02 (0.71-1.48)  0.85 (0.65-1.11) | 0.32 |  |
| Sexually active^3,4^  No  Yes | | 118 2540 4.6  242 6808 3.6 | 1 (reference)  0.77 (0.61-0.95) | 0.017 | 1 (reference)  0.79 (0.61-1.03) | 0.088 |
| Prior AIDS diagnosis^3,4^  No  Yes | | 283 7260 3.9  77 2088 3.7 | 1 (reference)  0.95 (0.73-1.21) | 0.67 | 1 (reference)  0.97 (0.72-1.31) | 0.84 |
| CD4 cell nadir [cells/µL]^3,4^  350+  200-349  100-199  <100 | | 119 3215 3.7  74 1406 5.3  84 2269 3.7  83 2459 3.4 | 1 (reference)  0.70 (0.53-0.94)  0.70 (0.51-0.96)  0.64 (0.47-0.88) | 0.029  0.021^2^ | 1 (reference)  0.68 (0.50-0.93)  0.69 (0.49-0.96)  0.55 (0.37-0.80) | 0.018  <0.008^2^ |
| ART and viral suppression^3,4^  On ART, VL <50 copies/mL  On ART, VL >50 copies/mL  Not on ART | | 307 8168 3.8  26 622 4.2  27 558 4.8 | 1 (reference)  1.11 (0.75-1.66)  1.29 (0.87-1.91) | 0.41 | 1 (reference)  1.02 (0.68-1.52)  0.94 (0.61-1.45) | 0.95 |
| Active HCV infection^3,4^  No  Yes | | 314 8620 3.6  46 728 6.3 | 1 (reference)  1.74 (1.27-2.37) | <0.001 | 1 (reference)  1.44 (0.91-2.28) | 0.12 |
| Active HBV infection^3,4^  No  Yes | | 347 8924 3.9  13 424 3.1 | 1 (reference)  0.79 (0.45-1.38) | 0.40 | 1 (reference)  0.74 (0.41-1.31) | 0.30 |

| BMI [kg/m^2^]^3,4^  <18.5  18.5-24.9  25-29.9  30+ | 22 342 6.4  200 5402 3.7  107 2824 3.8  31 781 4.0 | 1.74 (1.12-2.70)  1 (reference)  1.02 (0.81-1.29)  1.07 (0.73-1.57) | 0.10 | 1.28 (0.81-2.02)  1 (reference)  1.14 (0.90-1.45)  1.13 (0.76-1.69) | 0.56 |
| --- | --- | --- | --- | --- | --- |
| Current injection drug use^3,4^  No  Yes | 354 9293 3.8  6 55 10.9 | 1 (reference)  2.87 (1.30-6.36) | 0.009 | 1 (reference)  1.21 (0.47-3.13) | 0.70 |
| Cocaine (non-injection)^3,4^  No  Yes | 339 9054 3.7  21 294 7.1 | 1 (reference)  1.91 (1.23-2.96) | 0.004 | 1 (reference)  1.52 (0.89-2.60) | 0.13 |
| Other non-injection drugs^3,4^  No  Yes | 343 9030 3.8  17 318 5.4 | 1 (reference)  1.41 (0.86-2.30) | 0.17 | 1 (reference)  0.96 (0.54-1.72) | 0.90 |

^1^ P-values from Poisson regression unless indicated otherwise,

^2^ P-values from Poisson regression testing for trend across groups

^3^ Variable has been time-updated,

^4^ Variable has been lagged for 90 days

Abbreviations: IR, incidence rate per 100 PY; IRR, incidence rate ratio; CI, confidence interval; PY, person years of follow-up; MSM, men who have sex with men; HET, heterosexual transmission; IDU, injection drug use; ART, antiretroviral therapy; VL, HIV viral load; HBV, hepatits B virus; HCV, hepatitis C virus; BMI, body mass index.
