# Supplementary material for: Frequency of and Risk Factors for Depression among Participants in the Swiss HIV Cohort Study (SHCS)
Source: PLoS One. 2015 Oct 22;10(10):e0140943. doi: 10.1371/journal.pone.0140943 (PMC4619594; doi:10.1371/journal.pone.0140943)
Supplement: S2 Table — (DOCX) [file pone.0140943.s002.docx]

**Table S2: Sensitivity analyses: Poisson regression analysis of risk for incident depression among 3,990 non-IDU cohort participants free of depression at the first two baseline visits and without a history of prior psychiatric disorders.**

| Characteristic | Events PY IR | Univariable analyses  IRR (95% CI) | P-value^1^ | Multivariable analysis  IRR (95% CI) | P-value^1^ |
| --- | --- | --- | --- | --- | --- |
| Total | 310 8459 3.7 |  |  |  |  |
| Risk group  White MSM  White male HET  White female HET  Non-white male  Non-white female | 143 4117 3.5  39 1386 2.8  51 894 5.7  36 885 4.1  41 1177 3.5 | 1 (reference)  0.81 (0.57-1.15)  1.64 (1.19-2.26)  1.17 (0.81-1.69)  1.00 (0.71-1.42) | 0.009 | 1 (reference)  0.87 (0.61-1.26)  1.61 (1.14-2.28)  1.17 (0.80-1.72)  0.91 (0.60-1.38) | 0.031 |
| Age [years]^3^  <45  45-54  55+ | 152 3593 4.2  112 3077 3.6  46 1789 2.6 | 1 (reference)  0.86 (0.67-1.10)  0.61 (0.44-0.85) | 0.012  0.003^2^ | 1 (reference)  0.94 (0.73-1.22)  0.62 (0.42-0.90) | 0.031  0.020^2^ |
| Alcohol consumption^3,4^  None  Light  Moderate/heavy | 158 3514 4.5  134 4497 3.0  18 448 4.0 | 1 (reference)  0.66 (0.53-0.83)  0.89 (0.55-1.45) | 0.002 | 1 (reference)  0.70 (0.54-0.90)  0.81 (0.48-1.37) | 0.015 |
| Smoking  No  Yes, without cannabis use  Yes, including cannabis use | 185 5594 3.3  80 2230 3.6  45 635 7.1 | 0.92 (0.71-1.20)  1 (reference)  1.97 (1.37-2.85) | <0.001 | 1.00 (0.75-1.33)  1 (reference)  1.82 (1.23-2.70) | 0.006 |
| Activity [30 min./day]^3,4^  None  <1/week  >1/week | 143 3650 3.9  39 1001 3.9  128 3808 3.4 | 1 (reference) 0.99 (0.70-1.42)  0.86 (0.68-1.09) | 0.42  0.021^2^ | 1 (reference)  0.99 (0.69-1.42)  0.83 (0.64-1.07) | 0.29  0.15^2^ |
| Ability to work [%]^3,4^  <50  50-74  75+ (full) | 34 752 4.5  25 322 4.7  261 7385 3.5 | 1 (reference)  1.03 (0.56-1.89)  0.78 (0.55-1.12) | 0.26 | 1 (reference)  0.95 (0.51-1.77)  0.68 (0.45-1.03) | 0.13 |

| Living situation^3,4^  Alone, single  Alone, partner  Not alone | 87 2186 4.0  40 1023 3.9  183 5250 3.5 | 1 (reference)  0.98 (0.68-1.43)  0.88 (0.68-1.13) | 0.54 | 1 (reference)  1.00 (0.68-1.49)  0.88 (0.66-1.17) | 0.59 |  |
| --- | --- | --- | --- | --- | --- | --- |
| Sexually active^3,4^  No  Yes | | 91 2249 4.0  219 6209 3.5 | 1 (reference)  0.87 (0.68-1.11) | 0.27 | 1 (reference)  0.85 (0.64-1.15) | 0.30 |
| Prior AIDS diagnosis^3,4^  No  Yes | | 248 6655 3.7  62 1804 3.4 | 1 (reference)  0.92 (0.70-1.22) | 0.57 | 1 (reference)  1.02 (0.72-1.43) | 0.93 |
| CD4 cell nadir [cells/µL]^3,4^  350+  200-349  100-199  <100 | | 109 2972 3.7  86 1327 5.1  68 2056 3.3  65 2104 3.1 | 1 (reference)  0.72 (0.53-0.97)  0.65 (0.46-0.90)  0.60 (0.43-0.85) | 0.017  0.006^2^ | 1 (reference)  0.71 (0.52-0.99)  0.67 (0.46-0.97)  0.55 (0.36-0.84) | 0.040  0.011^2^ |
| ART and viral suppression^3,4^  On ART, VL <50 copies/mL  On ART, VL >50 copies/mL  Not on ART | | 263 7389 3.6  22 557 4.0  25 513 4.9 | 1 (reference)  1.11 (0.72-1.72)  1.37 (0.91-2.06) | 0.31 | 1 (reference)  1.02 (0.66-1.58)  0.99 (0.63-1.55) | 0.99 |
| Active HCV infection^3,4^  No  Yes | | 293 8232 3.6  17 227 7.5 | 1 (reference)  2.11 (1.28-3.48) | 0.003 | 1 (reference)  1.87 (1.11-3.16) | 0.019 |
| Active HBV infection^3,4^  No  Yes | | 300 8088 3.7  10 370 2.7 | 1 (reference)  0.73 (0.39-1.37) | 0.32 | 1 (reference)  0.65 (0.33-1.30) | 0.23 |
| BMI [kg/m^2^]^3,4^  <18.5  18.5-24.9  25-29.9  30+ | | 14 243 5.8  172 4815 3.6  96 2659 3.6  28 742 3.8 | 1.61 (0.94-2.77)  1 (reference)  1.01 (0.79-1.30)  1.06 (0.70-1.58) | 0.38 | 1.29 (0.74-2.25)  1 (reference)  1.12 (0.87-1.44)  1.10 (0.73-1.68) | 0.72 |

| Current injection drug use^3,4^  No  Yes | 308 8455 3.6  2 3.7 53.8 | 1 (reference)  14.8 (2.95-73.9) | 0.001 | 1 (reference)  9.07 (1.48-55.7) | 0.017 |
| --- | --- | --- | --- | --- | --- |
| Cocaine (non-injection)^3,4^  No  Yes | 291 8199 3.5  19 260 7.3 | 1 (reference)  2.06 (1.29-3.27) | 0.002 | 1 (reference)  1.44 (0.76-2.74) | 0.27 |
| Other non-injection drugs^3,4^  No  Yes | 295 8194 3.6  15 265 5.7 | 1 (reference)  1.57 (0.94-2.65) | 0.087 | 1 (reference)  1.12 (0.58-2.18) | 0.73 |

^1^ P-values from Poisson regression unless indicated otherwise,

^2^ P-values from Poisson regression testing for trend across groups

^3^ Variable has been time-updated,

^4^ Variable has been lagged for 90 days

Abbreviations: IR, incidence rate per 100 PY; IRR, incidence rate ratio; CI, confidence interval; PY, person years of follow-up; MSM, men who have sex with men; HET, heterosexual transmission; IDU, injection drug use; ART, antiretroviral therapy; VL, HIV viral load; HBV, hepatits B virus; HCV, hepatitis C virus; BMI, body mass index.
