# Supplementary material for: Frequency of and Risk Factors for Depression among Participants in the Swiss HIV Cohort Study (SHCS)
Source: PLoS One. 2015 Oct 22;10(10):e0140943. doi: 10.1371/journal.pone.0140943 (PMC4619594; doi:10.1371/journal.pone.0140943)
Supplement: S3 Table — All variables are included in the multivariable model where correlations between IDU, HCV and several life-style variables make results difficult to interpret. (DOCX) [file pone.0140943.s003.docx]

**Table S3: Logistic regression of factors associated with cumulative prevalence of depression by the end of the observation period. All variables are included in the multivariable model where correlations between IDU, HCV and several life-style variables make results difficult to interpret.**

| Characteristic | Events total (%) | Univariable analyses  OR (95% CI) | P-value^1^ | Multivariable analysis  OR (95% CI) | P-value^1^ |
| --- | --- | --- | --- | --- | --- |
| Total | 1937 6756 28.7 |  |  |  |  |
| Risk group  White MSM  White male HET  White female HET  White male IDU  White female IDU  Non-white male  Non-white female | 728 2787 26.1  157 828 19.0  220 681 32.3  328 744 44.1  188 398 47.2  126 555 22.7  190 763 24.9 | 1 (reference)  0.66 (0.55-0.80)  1.35 (1.13-1.62)  2.23 (1.89-2.64)  2.53 (2.04-3.14)  0.83 (0.67-1.03)  0.94 (0.78-1.13) | <0.001 | 1 (reference)  0.67 (0.55-0.83)  1.16 (0.95-1.42)  1.02 (0.81-1.29)  1.07 (0.81-1.41)  0.85 (0.67-1.07)  0.79 (0.63-0.98) | <0.001 |
| Age [years]^3^  <45  45-54  55+ | 798 2510 31.8  849 2868 29.6  290 1378 21.0 | 1 (reference)  0.90 (0.80-1.01)  0.57 (0.49-0.67) | <0.001  <0.001^2^ | 1 (reference)  0.74 (0.64-0.84)  0.39 (0.32-0.47) | <0.001  <0.001^2^ |
| Alcohol consumption^3,4^  None  Light  Moderate/heavy | 1024 3180 32.2  727 3082 23.6  186 494 37.7 | 1 (reference)  0.65 (0.58-0.73)  1.27 (1.04-1.55) | <0.001 | 1 (reference)  0.81 (0.71-0.92)  1.11 (0.89-1.39) | <0.001 |
| Smoking  No  Yes, without cannabis use  Yes, including cannabis use | 847 2878 22.7  707 2096 33.7  383 935 41.0 | 0.58 (0.51-0.65)  1 (reference)  1.36 (1.16-1.60) | <0.001 | 0.80 (0.70-0.92)  1 (reference)  1.12 (0.94-1.34) | <0.001 |
| Activity [30minutes/day]^3,4^  None  <1/week  >1/week | 1055 3127 33.7  189 784 24.1  693 2845 24.4 | 1 (reference)  0.62 (0.52-0.75)  0.63 (0.56-0.71) | <0.001  <0.001^2^ | 1 (reference)  0.76 (0.63-0.93)  0.74 (0.65-0.84) | <0.001  <0.001^2^ |

| Ability to work [%]^3,4^  <50  50-74  75+ (full) | 687 1402 49.0  221 451 49.0  1029 4903 21.0 | 1 (reference)  1.00 (0.81-1.23)  0.28 (0.24-0.31) | <0.001 | 1 (reference)  1.01 (0.80-1.27)  0.27 (0.23-0.32) | <0.001 |
| --- | --- | --- | --- | --- | --- |
| Living situation^3,4^  Alone, single  Alone, partner  Not alone | 681 1958 34.8  243 839 29.0  1013 3959 25.6 | 1 (reference)  0.76 (0.64-0.91)  0.64 (0.57-0.72) | <0.001 | 1 (reference)  0.95 (0.78-1.16)  0.75 (0.65-0.86) | <0.001 |
| Sexually active^3,4^  No  Yes | 831 2284 36.4  1106 4472 24.7 | 1 (reference)  0.57 (0.52-0.64) | <0.001 | 1 (reference)  0.75 (0.65-0.86) | <0.001 |
| Prior AIDS diagnosis^3,4^  No  Yes | 1508 5190 29.1  429 1566 27.4 | 1 (reference)  0.92 (0.81-1.05) | 0.20 | 1 (reference)  0.81 (0.69-0.95) | 0.010 |
| CD4 cell nadir [cells/µL]^3,4^  350+  200-349  100-199  <100 | 314 993 31.6  643 2282 28.2  472 1661 28.4  508 1820 27.9 | 1 (reference)  0.84 (0.72-1.00)  0.86 (0.72-1.02)  0.84 (0.71-0.99) | 0.17  0.11^2^ | 1 (reference)  0.92 (0.76-1.10)  0.89 (0.73-1.09)  0.76 (0.61-0.94) | 0.053  0.009^2^ |
| ART and viral suppression^3,4^  On ART, VL <50 copies/mL  On ART, VL >50 copies/mL  Not on ART | 1551 5927 26.2  205 448 45.8  181 381 47.5 | 1 (reference)  2.38 (1.96-2.89)  2.55 (2.07-3.15) | <0.001 | 1 (reference)  2.11 (1.70-2.61)  2.56 (2.01-3.26) | <0.001 |
| Active HCV infection^3,4^  No  Yes | 1550 5856 26.5  387 900 43.0 | 1 (reference)  2.10 (1.81-2.42) | <0.001 | 1 (reference)  0.99 (0.80-1.22) | 0.92 |
| Active HBV infection^3,4^  No  Yes | 1840 6445 28.6  97 311 31.2 | 1 (reference)  1.13 (0.89-1.45) | 0.32 | 1 (reference)  1.08 (0.83-1.41) | 0.56 |

| BMI [kg/m^2^] ^3,4^  <18.5  18.5-24.9  25-29.9  30+ | 122 306 39.9  1129 3883 29.1  500 1932 25.9  186 635 29.3 | 1.62 (1.27-2.05)  1 (reference)  0.85 (0.75-0.96)  1.01 (0.84-1.22) | <0.001 | 0.95 (0.73-1.24) 1 (reference) 1.00 (0.87-1.14) 1.07 (0.87-1.32) | 0.88 |
| --- | --- | --- | --- | --- | --- |
| Current injection drug use^3,4^  No  Yes | 1869 6643 28.1  68 113 60.2 | 1 (reference)  3.86 (2.64-5.65) | 0.001 | 1 (reference)  1.28 (0.83-1.96) | 0.26 |
| Cocaine (non-injection)^3,4^  No  Yes | 1833 6492 28.2  104 264 39.4 | 1 (reference)  1.65 (1.28-2.13) | 0.001 | 1 (reference)  1.19 (0.88-1.61) | 0.27 |
| Other non-injection drugs^3,4^  No  Yes | 1813 6440 28.2  124 316 39.2 | 1 (reference)  1.65 (1.31-2.08) | <0.001 | 1 (reference)  1.12 (0.85-1.49) | 0.41 |

^1^ P-values from logistic regression unless indicated otherwise,

^2^ P-values from logistic regression testing for trend across groups

^3^ Variable has been time-updated,

^4^ Variable has been lagged for 90 days

Abbreviations: OR, Odds ratio; CI, confidence interval; PY, person years of follow-up; MSM, men who have sex with men; HET, heterosexual transmission; IDU, injection drug use; ART, antiretroviral therapy; VL, HIV viral load; HBV, hepatits B virus; HCV, hepatitis C virus; BMI, body mass index.
