# Supplementary material for: Frequency of and Risk Factors for Depression among Participants in the Swiss HIV Cohort Study (SHCS)
Source: PLoS One. 2015 Oct 22;10(10):e0140943. doi: 10.1371/journal.pone.0140943 (PMC4619594; doi:10.1371/journal.pone.0140943)
Supplement: S4 Table — All variables are included in the multivariable model. (DOCX) [file pone.0140943.s004.docx]

**Table S4: Sensitivity analyses: Logistic regression of factors associated with cumulative prevalence of depression among 5614 non-IDU participants by the end of the observation period. All variables are included in the multivariable model.**

| Characteristic | Events total (%) | Univariable analyses  OR (95% CI) | P-value^1^ | Multivariable analysis  OR (95% CI) | P-value^1^ |
| --- | --- | --- | --- | --- | --- |
| Total | 1421 5614 25.3 |  |  |  |  |
| Risk group  White MSM  White male HET  White female HET  Non-white male  Non-white female | 728 2787 26.1  157 828 19.0  220 681 32.3  126 555 22.7  190 763 24.9 | 1 (reference)  0.66 (0.55-0.80)  1.35 (1.13-1.62)  0.83 (0.67-1.03)  0.94 (0.78-1.13) | <0.001 | 1 (reference)  0.66 (0.53-0.82)  1.17 (0.95-1.43)  0.86 (0.68-1.09)  0.81 (0.65-1.01) | <0.001 |
| Age [years]^3^  <45  45-54  55+ | 620 2186 28.4  552 2181 25.3  249 1247 20.0 | 1 (reference)  0.86 (0.75-0.98)  0.63 (0.53-0.74) | <0.001  <0.001^2^ | 1 (reference)  0.77 (0.66-0.89)  0.39 (0.32-0.48) | <0.001  <0.001^2^ |
| Alcohol consumption^3,4^  None  Light  Moderate/heavy | 720 2558 28.2  585 2715 21.6  116 341 34.0 | 1 (reference)  0.70 (0.62-0.80)  1.32 (1.04-1.67) | <0.001 | 1 (reference)  0.82 (0.71-0.94)  1.29 (0.99-1.69) | <0.001 |
| Smoking  No  Yes, without cannabis use  Yes, including cannabis use | 776 3508 22.1  471 1575 29.9  174 531 32.8 | 0.67 (0.58-0.76)  1 (reference)  1.14 (0.93-1.41) | <0.001 | 0.80 (0.69-0.93)  1 (reference)  1.12 (0.89-1.41) | 0.001 |
| Activity [30minutes/day]^3,4^  None  <1/week  >1/week | 748 2504 29.9  150 681 22.0  523 2429 21.5 | 1 (reference)  0.66 (0.54-0.81)  0.64 (0.57-0.73) | <0.001  <0.001^2^ | 1 (reference)  0.77 (0.62-0.96)  0.71 (0.62-0.82) | <0.001  <0.001^2^ |
| Ability to work [%]^3,4^  <50  50-74  75+ (full) | 363 813 44.7  147 308 47.7  911 4493 20.3 | 1 (reference)  1.13 (0.87-1.47)  0.32 (0.27-0.37) | <0.001 | 1 (reference)  1.03 (0.77-1.36)  0.25 (0.21-0.30) | <0.001 |

| Living situation^3,4^  Alone, single  Alone, partner  Not alone | 497 1590 31.3  190 716 26.5  734 3308 22.2 | 1 (reference)  0.79 (0.65-0.97)  0.63 (0.55-0.72) | <0.001 | 1 (reference)  0.96 (0.77-1.19)  0.71 (0.61-0.83) | <0.001 |
| --- | --- | --- | --- | --- | --- |
| Sexually active^3,4^  No  Yes | 545 1738 31.4  876 3876 22.6 | 1 (reference)  0.64 (0.56-0.73) | <0.001 | 1 (reference)  0.74 (0.63-0.86) | <0.001 |
| Prior AIDS diagnosis^3,4^  No  Yes | 1110 4375 25.4  311 1239 25.1 | 1 (reference)  0.99 (0.85-1.14) | 0.85 | 1 (reference)  0.96 (0.80-1.15) | 0.64 |
| CD4 cell nadir [cells/µL]^3,4^  350+  200-349  100-199  <100 | 506 1979 25.6  270 906 29.8  318 1344 23.7  327 1385 23.6 | 1 (reference)  0.81 (0.68-0.96)  0.73 (0.60-0.88)  0.73 (0.60-0.88) | 0.004  0.001^2^ | 1 (reference)  0.93 (0.76-1.13)  0.83 (0.67-1.04)  0.67 (0.53-0.86) | 0.005  0.001^2^ |
| ART and viral suppression^3,4^  On ART, HIV <50 copies/mL  On ART, HIV >50 copies/mL  Not on ART | 1124 4934 22.8  154 356 43.3  143 324 44.1 | 1 (reference)  2.58 (2.07-3.22)  2.68 (2.13-3.37) | <0.001 | 1 (reference)  2.42 (1.91-3.07)  2.63 (2.02-3.42) | <0.001 |
| Active HCV infection^3,4^  No  Yes | 1358 5418 25.1  63 196 32.1 | 1 (reference)  1.42 (1.04-1.92) | 0.029 | 1 (reference)  1.29 (0.92-1.81) | 0.13 |
| Active HBV infection^3,4^  No  Yes | 1350 5359 25.2  71 255 27.8 | 1 (reference)  1.15 (0.87-1.52) | 0.35 | 1 (reference)  1.06 (0.78-1.43) | 0.72 |
| BMI [kg/m^2^] ^3,4^  <18.5  18.5-24.9  25-29.9  30+ | 62 185 33.5  810 3163 25.6  399 1703 23.4  150 563 26.6 | 1.46 (1.07-2.01)  1 (reference)  0.89 (0.77-1.02)  1.06 (0.86-1.29) | 0.018 | 0.94 (0.66-1.33)  1 (reference)  0.98 (0.84-1.14)  1.02 (0.81-1.28) | 0.97 |

| Current injection drug use^3,4^  No  Yes | 1416 5607 25.3  5 7 71.4 | 1 (reference)  7.40 (1.43-38.2) | 0.011 | 1 (reference)  4.84 (0.86-27.2) | 0.073 |
| --- | --- | --- | --- | --- | --- |
| Cocaine (non-injection)^3,4^  No  Yes | 1352 5417 25.0  69 197 35.0 | 1 (reference)  1.62 (1.20-2.19) | 0.002 | 1 (reference)  1.42 (1.00-2.03) | 0.052 |
| Other non-injection drugs^3,4^  No  Yes | 1359 5401 25.2  62 213 29.1 | 1 (reference)  1.22 (0.90-1.65) | 0.20 | 1 (reference)  0.92 (0.64-1.32) | 0.66 |

^1^ P-values from logistic regression unless indicated otherwise,

^2^ P-values from logistic regression testing for trend across groups

^3^ Variable has been time-updated,

^4^ Variable has been lagged for 90 days

Abbreviations: OR, Odds ratio; CI, confidence interval; PY, person years of follow-up; MSM, men who have sex with men; HET, heterosexual transmission; IDU, injection drug use; ART, antiretroviral therapy; VL, HIV viral load; HBV, hepatits B virus; HCV, hepatitis C virus; BMI, body mass index.
